# Metformin Mitigates Fibrosis and Glucose Intolerance Induced by Doxorubicin in Subcutaneous Adipose Tissue

**DOI:** 10.3389/fphar.2018.00452

**Published:** 2018-05-08

**Authors:** Luana A. Biondo, Helena A. Batatinha, Camila O. Souza, Alexandre A. S. Teixeira, Loreana S. Silveira, Maria I. Alonso-Vale, Lila M. Oyama, Michele J. Alves, Marilia Seelaender, José C. R. Neto

**Affiliations:** ^1^Department of Cellular and Developmental Biology, Institute of Biomedical Sciences, University of São Paulo (USP), São Paulo, Brazil; ^2^Exercise and Immunometabolism Research Group, Department of Physical Education, Universidade Estadual Paulista (UNESP), São Paulo, Brazil; ^3^Department of Biological Sciences, Institute of Environmental Sciences, Chemical and Pharmaceutical Sciences, Federal University of São Paulo (UNIFESP), São Paulo, Brazil; ^4^Department of Physiology, Physiology of Nutrition Discipline, Federal University of São Paulo (UNIFESP), São Paulo, Brazil; ^5^Department of Surgery, Faculty of Medicine, University of São Paulo (USP), São Paulo, Brazil

**Keywords:** doxorubicin, adipose tissue, metformin, fibrosis, chemotherapy, glucose

## Abstract

Doxorubicin (DX) is a chemotherapeutic drug that is used in clinical practice that promotes deleterious side effects in non-tumor tissues such as adipose tissue. We showed that DX leads to extensive damage in adipose tissue via a disruption in 5′-adenosine monophosphate-activated protein kinase (AMPK) and PPAR-gamma signaling. Thus, we investigated whether co-treatment with the biguanide drug metformin (MET) could prevent the side effects of DX through the activation of AMPK in adipose tissue. The goal of the present study was to verify the effects of DX and adjuvant MET treatment in subcutaneous adipose tissue (SAT) and to determine whether MET could protect against chemotherapy-induced side effects. C57/BL6 mice received DX hydrochloride (2.5 mg/kg) intraperitoneally 2 times per week for 2 weeks (DX), concomitantly or not, with MET administration (300 mg/kg oral daily) (DX + MET). The control group (CTRL) was pair-fed according to the food consumption of the DX group. After euthanasia, adipose tissue fat pads were collected, and SAT was extracted so that adipocytes could be isolated. Glucose uptake was then measured, and histological, gene, and protein analyses were performed. One-way analysis of variance was also performed, and significance was set to 5%. DX reduced retroperitoneal fat mass and epididymal pads and decreased glycemia. In cultured primary subcutaneous adipocytes, mice in the DX group had lower glucose uptake when stimulated with insulin compared with mice in the CTRL group. Adipocytes in the DX group exhibited a reduced area, perimeter, and diameter; decreased adiponectin secretion; and decreased fatty acid synthase gene expression. SAT from MET-treated mice also showed a reduction in collagen deposition. Treatment with MET prevented fibrosis and restored glucose uptake in SAT after insulin stimulation, yet the drug was unable to prevent other side effects of DX such as tissue loss and inflammatory response.

## Introduction

Doxorubicin is a chemotherapeutic belonging to the anthracycline family that is highly effective in the treatment of various adult and pediatric cancers such as lymphoma, solid tumors, leukemia, and prostate and breast cancer (Sun et al., 2016). Besides its efficacy, doxorubicin treatment decreases quality of life because of its side effects including vomiting, diarrhea, bleeding, extreme fatigue, discomfort, and anorexia, which contribute to patient debilitation (Gorselink et al., 2006; Hydock et al., 2011). Nevertheless, doxorubicin is known to promote severe toxic effects in non-tumor tissues; cardiotoxicity is well described in the literature (Benjamin et al., 1974; Hydock et al., 2011) as are nephrotoxicity (Singla et al., 2014) and hepatotoxicity (Gorselink et al., 2006; Ayla et al., 2011).

Although many researchers have demonstrated the toxic effects of doxorubicin on cardiac muscle, few studies have investigated the side effects of doxorubicin on adipose tissue. The loss of adipose tissue and skeletal muscle after chemotherapy are associated with a worse prognosis and a reduction in quality of life (Rutten et al., 2016) and can lead to metabolic and physiological disturbances. White adipose tissue is an endocrine organ that secretes a variety of adipokines that promotes cross-talk within diverse organs (e.g., heart, muscle, and bone marrow). Adipokines are tightly associated with energy balance (Argilés et al., 2005; Ghadge et al., 2014). For example, adiponectin regulates lipid and glucose metabolism (Smitka and Marešová, 2015), whereas leptin modulates ingestion and energy expenditure (Szewczyk-Golec et al., 2015). Therefore, the accelerated loss of adipose mass can lead to disturbances in whole-body inflammatory and metabolic responses in cancer patients. Our group showed that a single dose of doxorubicin (15 mg/kg body weight) causes rapid loss of adipose mass (72 h) together with an imbalance in adipokines (Biondo et al., 2016).

In response to injury, for the repair of tissue damage, healing generally begins with local inflammation followed by tissue remodeling through the alteration of extracellular matrix components (Rockey et al., 2015). When a change in fat mass occurs, adipose tissue also undergoes structural remodeling. Excessive accumulation of fibrous connective tissue modifies the function of adipose tissue (Rockey et al., 2015; Chabot et al., 2017). High deposition of collagen, a major protein component of the extracellular matrix, is often associated with fibrosis (Divoux and Clément, 2011). In obesity, there is marked fibrosis of subcutaneous adipose tissue (SAT) (Divoux and Clément, 2011; Choe et al., 2016), but few studies have evaluated fibrosis associated with the loss of adipose tissue mass (Chabot et al., 2017). It is known that hospitalized cancer patients exhibit adipose tissue loss as a result of cancer-related cachexia (Argilés et al., 2015) and fibrosis.

Therefore, co-therapies, which may reduce the toxic effects of doxorubicin in white adipose tissue, can improve treatment efficacy and help to ameliorate the quality of life during treatment. We showed that doxorubicin decreased the activity of the two primary metabolic pathways of adipose tissue: the adenosine monophosphate-activated protein kinase (AMPK) and PPAR-gamma pathways. These alterations can lead to a disruption in metabolic reprogramming in adipose tissue. Metformin (MET) is a hypoglycemic drug that acts through AMPK activation and functions by limiting the synthesis of lipids and glucose and by inhibiting complex 1 of the electron transport chain during cellular respiration (Hawley et al., 2010; Viollet et al., 2012). Many studies demonstrated efficient results with this drug as a cancer treatment in both experimental and clinical practices (Hosono et al., 2010; Landman et al., 2010; Hadad et al., 2015; Kelleni et al., 2015) and have shown that MET co-treatment is efficacious against cancer. Regarding the cardiotoxicity induced by doxorubicin, it is known that MET treatment decreases the expression of proinflammatory enzymes (Kelleni et al., 2015), which induces AMPK signaling (Kobashigawa et al., 2014). Moreover, with respect to adipose tissue, MET induces the AMPK and PPAR signaling pathways and mitigates SAT fibrosis in older humans (Kulkarni et al., 2018).

Thus, the aim of this study was to verify the effects of doxorubicin and adjuvant MET treatment on SAT and to determine whether MET could mitigate the side effects of doxorubicin.

## Materials and Methods

### Animals

The Experimental Research Committee of the University of São Paulo approved all procedures for the care of the animals used in this study. All experiments were performed in accordance with the approved guidelines of the ICB-USP Animal Ethics Committee (registered under n°5, fls 15, book 03 of this Institute).

In all, 36 male C57/BL6 mice approximately 8 weeks of age (weighing 19–26 g) were obtained from the Biomedical Sciences Institute of the University of São Paulo and were housed 2 or 3 per cage in an animal room under a 12:12 h light–dark cycle.

**FIGURE 1:**
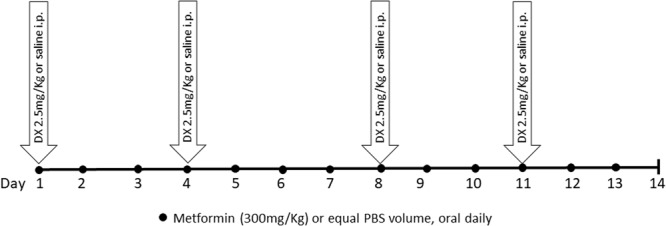
Experimental protocol design.

Mice were randomly divided into three groups: the control group (CTRL); the doxorubicin group (DX), in which mice were injected with doxorubicin hydrochloride at a dose of 2.5 mg/kg of body weight intraperitoneally (i.p.) twice a week for 2 weeks with (Eurofarma Laboratory, Campinas, São Paulo, Brazil); the doxorubicin and MET group (DX + MET), in which mice received doxorubicin i.p. and daily MET (Sigma-Aldrich, St. Louis, MO, United States) orally at a dose of 300 mg/kg of body weight. After the acclimation period, mice received a total of 10 mg/kg of doxorubicin, whereas the CTRL animals received an equal volume of saline (**Figure 1**).

The mice received food (chow pellet diet, Nuvilab CR1, Nuvital S/A, Colombo, Paraná, Brazil) and water. CTRL mice were pair-fed in accordance with the food consumption of the DX group; the food intake was equivalent among the groups to avoid any effects of differences in total calorie intake. Food intake and body weight were assessed every 2 and 3–4 days, respectively. After MET gavage, the health and well-being of the animals were monitored daily.

A total of 24 h after the last dose of MET, the mice were euthanized by decapitation, and SAT was removed, weighed, flash-frozen in liquid nitrogen, and stored at -80°C. Blood was drawn and centrifuged, and the serum was removed and kept frozen at -80°C for further analysis.

### Adipocyte Isolation

The subcutaneous fat pad was dissociated in Dulbecco’s Modified Eagle Medium (pH 7.4) (Sigma-Aldrich, St. Louis, MO, United States) supplemented with HEPES (20 mM), sodium pyruvate (2 mM), bovine serum albumin (BSA, 1%), and collagenase type II (1 mg/mL) in an orbital bath shaker at 37°C for 20 min. Isolated adipocytes were filtered and washed three times in the same buffer without collagenase. The adipocytes were observed under an optical microscope (×100 magnification) with an attached camera (Moticam 1000; Motic, Richmond, BC, Canada).

### 2-Deoxy-D-Glucose (2-DG) Uptake

Primary subcutaneous adipocytes (10^6^ cells/mL) were washed in PBS and incubated with or without insulin (100 nmol/L) in buffer containing 140 mM NaCl, 20 mM HEPES, 5 mM KCl, 2.5 mM MgSO_4_, 1 mM CaCl_2_, and 1% BSA (pH 7.4) for 20 min at 37°C. Subsequently, 2-deoxy-D-[3H]-glucose (0.4 mmol/L, 1850 Bq/tube or well) (Amersham Bioscience, United Kingdom) was added, and the reaction was allowed to occur for exactly 3 min. The reaction was interrupted by the addition of 250 μL ice-cold phloretin (0.3 mmol/L in Earle’s salts, 10 mM HEPES, 1% BSA, and 0.05% DMSO). The primary adipocytes and 200 μL of this final mixture were layered with 200 μL of silicone oil (density of 0.963 mg/mL) in microfuge tubes and were centrifuged for 10 s at 11,000*g*. The cell pellet on top of the oil layer was collected and transferred to vials containing the scintillation cocktail for radioactivity measurement using a Beta counter (450 LSC, Counter Beta, Trilux, Perkin Elmer). The results were normalized by diameter of the primary adipocytes and expressed as pmol per cm^2^.

### Subcutaneous Adipose Tissue Analysis: Adiponectin, IL-4, IL-8, IL-12, IFNg, and MCP-1

Frozen tissues (0.1 g) were homogenized in RIPA buffer (0.625% Nonidet P-40, 0.625% sodium deoxycholate, 6.25 mM sodium phosphate, and 1 M methylene-diaminetetraacetic acid at pH 7.4) containing 10 μg/mL of protease inhibitor cocktail (Sigma-Aldrich, St. Louis, MO, United States). Homogenates were centrifuged, the supernatant was recovered, and the protein concentration was determined using a Bradford assay (Bio-Rad, Hercules, CA, United States) with BSA as a reference. The quantitative assessment of adiponectin; interleukin (IL)-4, IL-8, and IL-12; interferon gamma (IFNg); and monocyte chemoattractant protein 1 (MCP-1) in SAT was performed by DuoSet Enzyme-Linked Immunosorbent Assay (DuoSet ELISA, R&D Systems, Minneapolis, MN, United States).

### Serum Analysis: Adiponectin, Leptin, and Lipopolysaccharides

The quantitative assessment of adiponectin and leptin in the serum was performed by ELISA (DuoSet ELISA, R&D Systems, Minneapolis, MN, United States). Lipopolysaccharides (LPS) were assessed by a QCL-1000 kit (Lonza, ref. 50-647U e 50-648U, Walkersville, MD, United States).

### Lipid Profile and Glucose Level

Serum levels of total cholesterol and triacylglycerol as well as glycemia were determined by enzymatic methods (Labtest, Lagoa Santa, Minas Gerais, Brazil). A colorimetric method assay was employed for the quantitative determination of non-esterified fatty acids in the serum (NEFA-HR, Wako, Mountain View, CA, United States).

### Histological Analyses

Small fragments of SAT were fixed in paraformaldehyde (10%), embedded in paraffin, and serially cross-sectioned into 5-μm-thick sections. The sections were stained with hematoxylin and eosin (H&E) for morphological analyses, and Picro Sirius Red for the detection of fibrosis. The 40× digital images were captured using an optical microscope with an attached AxioCamm HRC (Carl Zeiss, Andrade Gutierrez, Brazil). Quantification of the adipocyte area, perimeter, and diameter was performed using the 40× digital images of five independent sections obtained from each mouse. Fibrotic areas were expressed as a percentage compared with the total amount of tissue within the images.

### Quantitative Real-Time PCR

Total RNA was extracted from SAT with TRIzol (Invitrogen Life Technologies, Carlsbad, CA, United States) and reverse transcribed to cDNA using a High-Capacity cDNA kit (Applied Biosystems, Warrington, United Kingdom). Expression of genes related to glucose and lipid metabolism was evaluated by real-time PCR using a Rotor-Gene Q cycler (Qiagen) and SYBR Green as the fluorescent dye. Quantification of gene expression was performed with *RPL-19* gene as a control. The primer sequences are shown in **Table 1**.

**Table 1:** Primer sequences.

Gene	5′-Forward Primer-3′	5′-Reverse Primer-3′
*RPL-19*	CAATGCCAACTCCCGTCA	GTGTTTTTCCGGCAACGAG
*Fas*	GATTCGGTGTATCCTGCTGTC	CATGCTTTAGCACCTGCTGT
*Acc*	CCAGCAGATTGCCAACATC	ACTTCGGTACCTCTGCACCA
*Cebp*	TAGGTTTCTGGGCTTTGTGG	GATGGATCGATTGTGCTTCA
*Oct-1*	CCTGAAGATGATGTGCCTTG	ATCAGGATGAGGGTGTGCTT
*Col6a3*	GCTGCGGAATCACTTTGTGC	CACCTTGACACCTTTCTGGGT
*Fibronectin-1*	ATGTGGACCCCTCCTGATAGT	GCCCAGTGATTTCAGCAAAGG

### Statistical Analysis

Statistical analysis was performed using the GraphPad Prism statistics software package version 5.0 for Windows (GraphPad Software, San Diego, CA, United States). The data are expressed as means ± SD. Data were analyzed using the one-way analysis of variance (ANOVA) test with Bonferroni’s *post hoc* test. Values of *p* < 0.05^∗^, *p* < 0.01^∗∗^, *p* < 0.001^∗∗∗^, and *p* < 0.0001^∗∗∗∗^ were considered statistically significant.

Although each treatment group consisted of 12 mice, the amount of adipose tissue obtained from each individual was not sufficient to perform all experiments on all the mice. For this reason, some tests were performed on adipose tissue from a smaller subgroup as indicated in the figure captions.

## Results

**FIGURE 2:**
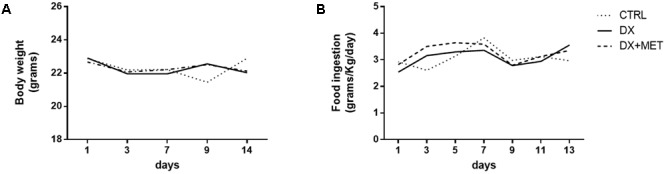
Body weight **(A)** and food consumption **(B)**: mice were injected with doxorubicin hydrochloride (DX) or saline (CTRL) i.p. twice a week for 2 weeks; a subset also received a daily oral gavage of metformin (DX + MET). Data are presented as means (*n* = 11). The groups were compared using one-way ANOVA followed by Bonferroni’s *post hoc* tests.

**FIGURE 3:**
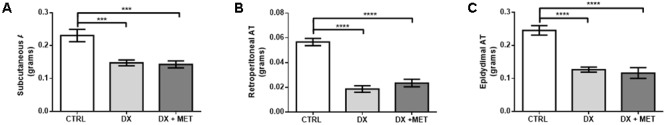
Weight of subcutaneous AT **(A)**, retroperitoneal AT **(B)**, and epididymal AT **(C)**: mice were injected with doxorubicin hydrochloride (DX) or saline (CTRL) i.p. twice a week for 2 weeks, and a subset received a daily oral gavage of metformin (DX + MET). Data are presented as means ± SD (*n* = 11). The groups were compared using one-way ANOVA followed by Bonferroni’s *post hoc* tests ^∗∗∗^*p* ≤ 0.001 and ^∗∗∗∗^*p* ≤ 0.0001.

Treatment for 2 weeks with doxorubicin and MET did not change the body weight or food ingestion of the mice (**Figures 2A,B**). However, doxorubicin treatment resulted in a reduction of subcutaneous, retroperitoneal, and epididymal adipose tissues depots (**Figures 3A–C**). MET treatment was not efficient in the prevention of adipose tissue loss.

**FIGURE 4:**
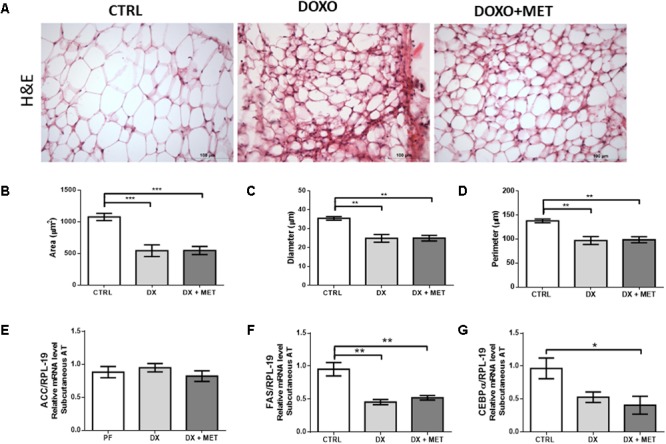
H&E staining **(A)** and the histological parameters of area **(B)**, diameter **(C)**, and perimeter of adipocytes **(D)**. Gene expression of acetyl coA carboxylase (*Acc*) **(E)**, fatty acid synthase (*Fas*) **(F)**, and CCAAT/enhancer-binding protein alpha (*Cebp*α) **(G)** in subcutaneous adipose tissue: mice were injected with doxorubicin hydrochloride (DX) or saline (CTRL) i.p. twice a week for 2 weeks, and a subset received a daily oral gavage of metformin (DX + MET). Data are presented as means ± SD (*n* = 5). The groups were compared using one-way ANOVA followed by Bonferroni’s *post hoc* tests ^∗^*p* < 0.05, ^∗∗^*p* < 0.005, and ^∗∗∗^*p* < 0.001.

In parallel with the decline in adipose tissue mass, doxorubicin promoted a reduction in area (**Figure 4B**), diameter (**Figure 4C**), and perimeter (**Figure 4D**) of adipocytes, that were shown in histological images (**Figure 4A**). These parameters were not restored by MET. Doxorubicin also reduced gene expression of *Fas* (**Figure 4E**), which is an essential enzyme for lipogenesis, but DX did not affect the mRNA expression of *Acc* and *Cebp*α (**Figures 4F,G**).

**FIGURE 5:**
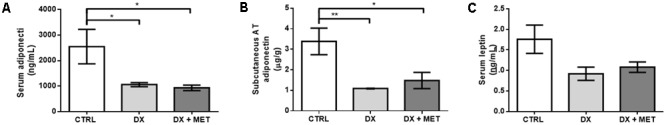
Serum adiponectin **(A)**, subcutaneous AT adiponectin **(B)**, and serum leptin **(C)**: mice were injected with doxorubicin hydrochloride (DX) or saline (CTRL) i.p. twice a week for 2 weeks, and a subset received a daily oral gavage of metformin (DX + MET). Data are presented as means ± SD (*n* = 4–5). The groups were compared using one-way ANOVA followed by Bonferroni’s *post hoc* tests ^∗^*p* ≤ 0.05 and ^∗∗^*p* ≤ 0.005.

**FIGURE 6:**
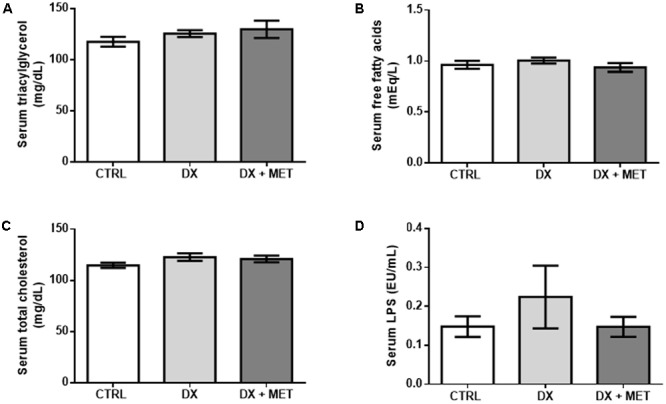
Triacylglycerol **(A)**, free fatty acids **(B)**, total cholesterol **(C)**, and LPS **(D)** in the serum: mice were injected with doxorubicin hydrochloride (DX) or saline (CTRL) i.p. twice a week for 2 weeks, and a subset received a daily oral gavage of metformin (DX + MET). Data are presented as means ± SD (*n* = 6–7). The groups were compared using one-way ANOVA followed by Bonferroni’s *post hoc* tests.

Doxorubicin treatment led to a reduction in adiponectin in the SAT and in the serum (**Figures 5A–C**). No changes were observed in the serum levels of leptin, triacylglycerol, free fatty acids, total cholesterol, and endotoxin (**Figures 6A–D**, respectively). Co-therapy with MET did not prevent alterations in the levels of adipokines, changes in the lipid profile, or changes in endotoxin concentration.

**FIGURE 7:**
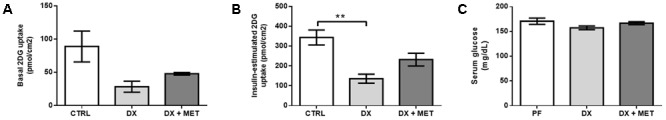
2-DG uptake in adipocytes extracted from the subcutaneous AT under basal conditions **(A)** and when stimulated by insulin **(B)**, and serum glucose level **(C)**: mice were injected with doxorubicin hydrochloride (DX) or saline (CTRL) i.p. twice a week for 2 weeks, and a subset received a daily oral gavage of metformin (DX + MET). Data are presented as means ± SD. *n* = 3–5 **(A,B)** *n* = 6 **(C)**. The groups were compared using one-way ANOVA followed by Bonferroni posttests ^∗∗^*p* ≤ 0.005.

Doxorubicin reduced glycemia (**Figure 7A**) and glucose uptake by primary adipocytes isolated from SAT under basal conditions as well as in an insulin stimulated-state (**Figures 7B,C**, respectively).

**FIGURE 8:**
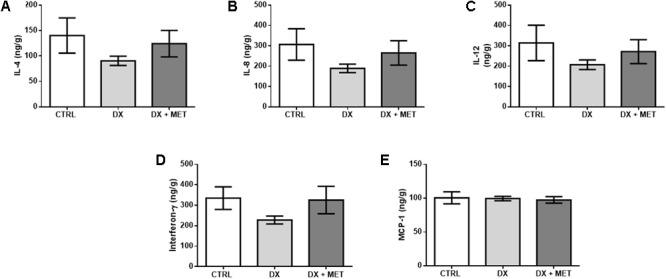
IL-4 **(A)**, IL-8 **(B)**, IL-12 **(C)**, interferon-gamma **(D)**, and MCP-1 in subcutaneous adipose tissue **(E)**: mice were injected with doxorubicin hydrochloride (DX) or saline (CTRL) i.p. twice a week for 2 weeks, and a subset received a daily oral gavage of metformin (DX + MET). Data are presented as means ± SD (*n* = 4). The groups were compared using one-way ANOVA followed by Bonferroni’s *post hoc* tests.

No drug effect was observed with respect to IL-4, IL-8, IL-12, IFNg, and MCP-1 concentrations (**Figures 8A–E**). In addition to the lack of statistical significance, these data suggest an extensive disruption of adipose tissue homeostasis by the chemotherapy drug DX and indicate that MET treatment did not prevent these effects.

**FIGURE 9:**
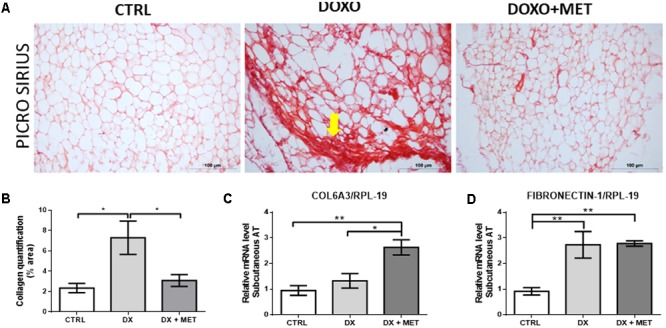
Picro Sirius Red staining **(A)**, collagen quantification **(B)**, gene expression of collagen 6 alpha 3 (*Col6a3*) **(C)**, and fibronectin-1 **(D)** in subcutaneous adipose tissue: mice were injected with doxorubicin hydrochloride (DX) or saline (CTRL) i.p. twice a week for 2 weeks, and a subset received a daily oral gavage of metformin (DX + MET). Data are presented as means ± SD (*n* = 4). The yellow arrow indicates fibrosis process. The groups were compared using one-way ANOVA followed by Bonferroni’s *post hoc* tests ^∗^*p* ≤ 0.05 and ^∗∗^*p* ≤ 0.005.

Finally, histological images showed that doxorubicin increased fibrosis in SAT and that MET treatment was efficient in preventing of this process (**Figures 9A,B**). Beyond that, the combined effect of the drugs elevated *Col6a3* gene expression (**Figures 9B,C**), and doxorubicin augmented fibronectin expression in both treatment groups (**Figures 9C,D**).

## Discussion

Doxorubicin administration is an effective treatment for several types of cancer and primarily functions through the promotion of cellular apoptosis (Rochette et al., 2015). However, this therapy has highly undesired side effects including, but not limited to, widely reported cardiotoxicity (Benjamin et al., 1974), DX also disrupts glucose and lipid metabolism and affects whole-body homeostasis (Biondo et al., 2016; De Lima Junior et al., 2016; Heart et al., 2016). We demonstrated that doxorubicin treatment caused a reduction in glucose uptake after insulin stimulation and caused marked fibrosis in SAT. Both side effects were partially counter-regulated by the co-administration of MET. However, MET treatment was unable to prevent adipose tissue atrophy and a reduction in adipokine production. Therefore, MET treatment may be employed for the attenuation of doxorubicin-induced side effects in SAT.

By itself, cancer leads to loss of adipose tissue mass, and chemotherapy only exacerbates this loss (Ebadi and Mazurak, 2014; Vergoni et al., 2016). Decrease in adipose mass contributes to a worse prognosis in cancer patients and is reported in some metabolic disorders such as cachexia and lipodystrophy (Ebadi and Mazurak, 2015). Our results showed a decrease in adipose tissue mass in different anatomical sites (retroperitoneal, subcutaneous, and epididymal) after doxorubicin treatment. In addition to the finding that body weight was maintained during the 2 weeks of treatment in all groups, the loss of adipose tissue mass was very significant in both the DX and the DX + MET groups. Because anorexia is a side effect of doxorubicin treatment, our control group was pair-fed according to the treatment groups. The weight of other organs and tissues analyzed (data not shown), such as muscle and liver, did not change. This way, the mice among the three groups have approximately the same body weight as a result of isoenergetic intake. In addition, the area, diameter, and perimeter of adipocytes from SAT were reduced by doxorubicin.

In accordance with the decline in adipose tissue mass and adipocyte dimensions, doxorubicin reduced the expression of the *Fas* gene; this gene encodes a key enzyme in the lipogenesis process, which is essential for the maintenance of adipose tissue mass. Together, these results showed an inhibition of SAT anabolic pathways (Brunengraber et al., 2003). Our group obtained similar findings in rats treated with a unique dose of doxorubicin (15 mg/kg body weight) (Biondo et al., 2016).

The endocrine function of adipose tissue was impaired, whereas the doxorubicin treatment caused reduction in adiponectin and total adiponectin in SAT. Low adiponectin is associated with the downregulation of PPAR-gamma gene expression; both effects were induced by doxorubicin and were shown in our previous study (Biondo et al., 2016). This impairment in adipokine homeostasis could induce changes in the physiological function of adipose tissue, including lipid mobilization factors and local and systemic inflammation. Treatment with MET induced the activation of PPARs in SAT of older adults (Kulkarni et al., 2018). However, in our study, MET was not able to restore adiponectin or *Fas* expression in SAT, which indicates that PPAR activity was not restored by co-administration of MET.

In general, adiponectin concentration is inversely associated with an insulin-resistant state (Weyer et al., 2001) through the AMPK pathway. MET is a potent AMPK activator drug in adipose tissue and promotes GLUT-4 translocation and, in this way, sensitizes cells to insulin (Lee et al., 2012). Our results showed that MET co-treatment restored stimulated glucose uptake by isolated adipocytes.

Disturbances promoted by doxorubicin administration could lead to higher expression of chemokines in adipose tissue and adipocytes (Vergoni et al., 2016). In contrast to these data, we did not observe any increase in inflammatory markers or chemoattractants of immune system cells in adipose tissue. It is probable that the administration of doxorubicin over 2 weeks was considered a chronic protocol that leads to a severe loss of adipose tissue, which could impair the secretion of chemokines and adipokines.

LPS is a glycolipid found in membrane of Gram-negative bacteria. In gut, it can increases the paracellular permeability, allowing the entrance of bacteria through gut epithelial cells to the serum. LPS activates toll-like receptor 4 in cells of immune system, such as macrophages and dendritic cells, and triggers inflammatory responses such as production of cytokines (Bein et al., 2017). The doxorubicin and MET did not change endotoxin concentrations and inflammatory markers.

Doxorubicin-induced fibrosis and inflammation have been described in different organs, such as the liver, heart, and kidney (Henninger et al., 2012; Ma et al., 2012; Szalay et al., 2015). Generally, both fibrosis and inflammation were found after a single high dose of doxorubicin (10 mg/kg body weight), which led to a reduction in survival rate (Krysko et al., 2011; Henninger et al., 2012; Ma et al., 2012). Our findings showed that low doses of doxorubicin (2.5 mg/kg body weight), as part of a chronic administration protocol (twice per week for 14 days), exacerbated fibrosis in the stromal vascular fraction and reduced the number of adipocytes. The co-administration of MET could reduce the extent of fibrosis and restore cell area. This anti-fibrotic effect of MET is well described in the literature in several tissue types (Lu et al., 2015; Doyle et al., 2016; Mummidi et al., 2016; Shen et al., 2016) including in adipose tissue of obese mice (Luo et al., 2016).

This increase in fibrosis leads to complications in adipose tissue such as high extracellular matrix rigidity and reduced expandability. It also induces ectopic lipid deposition, which leads to insulin resistance and impairment of metabolic pathways (Xie et al., 2017).

Unexpectedly, the mRNA expression of *Col6a3* was increased in the group treated with MET. This result was contradictory to the mitigation of fibrosis found in the DX + MET group. Moreover, fibronectin mRNA was increased in both the DX and the DX + MET groups. Elevation of fibronectin and *Col6a3* gene expression did not reveal the pathways that lead to fibrosis. We hypothesize that MET impairs the translation of these genes or that it augments collagen degradation, which would reduce collagen deposition. Recently, Kulkarni et al. (2018) showed that MET regulates the expression of genes that function in extracellular matrix organization and genes related to collagen chain trimerization in SAT of older humans. Thus, the molecular mechanism by which MET reduces fibrosis in SAT after doxorubicin treatment is still unclear, but the physiological and clinical relevance of this finding may improve the quality of life of patients.

Doxorubicin leads to cellular apoptosis in adipose tissue via the induction of DNA damage, as DX inhibits topoisomerase 2, which causes double-strand breaks in DNA, and consequently, cell death (Rochette et al., 2015). Additionally, researchers have used this medicine in mice and in cultured cell lines to induce apoptosis in adipose-derived cells through p53 activation (Vergoni et al., 2016). This way, a reduction in the availability of adipocytes and stromal vascular cells by doxorubicin could compromise gene transcription, which could cause this discrepancy between *Col6a3* and fibronectin mRNA in comparison with the total fibrotic area. In agreement with this, the co-incubation of MET an adipocyte culture treated with doxorubicin resulted in an increase in cell viability (data not shown).

## Conclusion

The administration of MET concomitant with doxorubicin treatment attenuated fibrosis and insulin resistance caused by this chemotherapy drug in SAT. MET showed significant results, which demonstrate the importance of new studies to clarify the role of MET in the reduction of chemotherapy-related side effects in relevant organs to metabolism control.

## Ethics Statement

We certify that the protocol registered under n°5 in fls 15 of book 03 for use of animals under experimentation, under the responsibility of JN, Coordinator of the Research Line “Effect of doxorubicin on metabolic disorders in adipose tissue and the possible adjuvant role of metformin,” in which the student(s) Edson Alves de Lima Júnior and LB, is in accordance with the Ethical Principles of Animal Experimentation adopted by the Brazilian Society of Laboratory Animal Science (SBCAL) and was approved by the Ethics Commission on Animal USE (CEUA) in March 10, 2014, valid for 4 years. São Paulo, March 12, 2014; Prof. Dr. Wothan Tavares De Lima, Coordinator – CEUA – ICB/USP; Prof. Dr. Ana Paula Lepique, Secretary – CEUA – ICB/USP.

## Author Contributions

LB, CS, HB, AT, and LS designed and performed the study, analyzed the results, and wrote the manuscript. MA-V and MA performed the experiments, analyzed the results, and revised the manuscript. LO and MS designed the study and revised the manuscript. JN designed the study, analyzed the results, revised the manuscript, and supervised the study. All authors read and approved the manuscript.

## Conflict of Interest Statement

The authors declare that the research was conducted in the absence of any commercial or financial relationships that could be construed as a potential conflict of interest.
